# Derivation and Validation of a Clinical Prediction Rule for Upper Limb Functional Outcomes After Traumatic Cervical Spinal Cord Injury

**DOI:** 10.1001/jamanetworkopen.2022.47949

**Published:** 2022-12-21

**Authors:** Saad Javeed, Jacob K. Greenberg, Justin K. Zhang, Christopher F. Dibble, Jawad M. Khalifeh, Ying Liu, Thomas J. Wilson, Lynda J. Yang, Yikyung Park, Wilson Z. Ray

**Affiliations:** 1 Department of Neurological Surgery, Washington University, St. Louis, Missouri; 2 Department of Neurological Surgery, Johns Hopkins University, Baltimore, Maryland; 3 Division of Public Health Sciences, Department of Surgery, Washington University School of Medicine, St Louis, Missouri; 4 Department of Neurosurgery, Stanford University, Stanford, California; 5 Department of Neurological Surgery, University of Michigan School of Medicine, Ann Arbor

## Abstract

**Importance:**

Traumatic cervical spinal cord injury (SCI) can result in debilitating paralysis. Following cervical SCI, accurate early prediction of upper limb recovery can serve an important role in guiding the appropriateness and timing of reconstructive therapies.

**Objective:**

To develop a clinical prediction rule to prognosticate upper limb functional recovery after cervical SCI.

**Design, Setting, and Participants:**

This prognostic study was a retrospective review of a longitudinal cohort study including patients enrolled in the National SCI model systems (SCIMS) database in US. Eligible patients were 15 years or older with tetraplegia (neurological level of injury C1-C8, American Spinal Cord Injury Association [ASIA] impairment scale [AIS] A-D), with early (within 1 month of SCI) and late (1-year follow-up) clinical examinations from 2011 to 2016. The data analysis was conducted from September 2021 to June 2022.

**Main Outcomes and Measures:**

The primary outcome was a composite of dependency in eating, bladder management, transfers, and locomotion domains of functional independence measure at 1-year follow-up. Each domain ranges from 1 to 7 with a lower score indicating greater functional dependence. Composite dependency was defined as a score of 4 or higher in at least 3 chosen domains. Multivariable logistic regression was used to predict the outcome based on early neurological variables. Discrimination was quantified using *C* statistics, and model performance was internally validated with bootstrapping and 10-fold cross-validation. The performance of the prediction score was compared with AIS grading. Data were split into derivation (2011-2014) and temporal-validation (2015-2016) cohorts.

**Results:**

Among 2373 patients with traumatic cervical SCI, 940 had complete 1-year outcome data (237 patients [25%] aged 60 years or older; 753 men [80%]). The primary outcome was present in 118 patients (13%), which included 92 men (78%), 83 (70%) patients who were younger than 60 years, and 73 (62%) patients experiencing AIS grade A SCI. The variables significantly associated with the outcome were age (age 60 years or older: OR, 2.31; 95% CI, 1.26-4.19), sex (men: OR, 0.60; 95% CI, 0.31-1.17), light-touch sensation at C5 (OR, 0.44; 95% CI, 0.44-1.01) and C8 (OR, 036; 95% CI, 0.24-0.53) dermatomes, and motor scores of the elbow flexors (C5) (OR, 0.74; 95% CI, 0.60-0.89) and wrist extensors (C6) (OR, 0.61; 95% CI, 0.49-0.75). A multivariable model including these variables had excellent discrimination in distinguishing dependent from independent patients in the temporal-validation cohort (*C* statistic, 0.90; 95% CI, 0.88-0.93). A clinical prediction score (range, 0 to 45 points) was developed based on these measures, with higher scores increasing the probability of dependency. The discrimination of the prediction score was significantly higher than from AIS grading (change in AUC, 0.14; 95% CI, 0.10-0.18; *P* < .001).

**Conclusions and Relevance:**

The findings of this study suggest that this prediction rule may help prognosticate upper limb function following cervical SCI. This tool can be used to set patient expectations, rehabilitation goals, and aid decision-making regarding the appropriateness and timing for upper limb reconstructive surgeries.

## Key Points

**Question:**

Can early neurological examination following traumatic cervical spinal cord injury predict upper limb functional outcomes at 1-year follow-up?

**Findings:**

In this prognostic study, a multivariable model was developed based on early clinical and neurological variables within 30 days of spinal cord injury. A model including age, sex, light touch scores at C5 and C8 dermatomes, and motor grades in elbow flexion (C5) and wrist extension (C6) predicted upper limb functional dependency 1 year after spinal cord injury.

**Meaning:**

These results suggest that use of this clinical prediction rule may guide patient counselling and clinical decision-making for reconstructive therapies in tetraplegia.

## Teaser

This prognostic study reports the development and validation of a clinical prediction rule that uses the results of a neurological examination to predict upper limb functional outcomes among patients with traumatic cervical spinal cord injury at 1-year follow-up.

## Introduction

Affecting over 17 900 people annually in the US, spinal cord injury (SCI) results in chronic impairment and disability.^1,2^ Recovery from a complete SCI is exceedingly rare, leaving most patients with permanent disability. Approximately 50% of SCI occurs at the cervical level, resulting in some loss of arm or hand function (ie, tetraplegia).^3^ Persons with tetraplegia rate restoration of hand function among their highest priorities.^4^ Regaining even partial arm or hand control can have a profound effect on functional independence and quality of life.

Among the rehabilitative therapies for tetraplegia, reconstructive upper limb surgery (ie, nerve and tendon transfers) remains the most reliable option for meaningful return of function.^5,6^ However, reconstructive upper limb surgeries carry risk, and subsets of SCI patients have significant potential for spontaneous recovery without any intervention.^7^ There are competing interests at play, whereby early surgery may impair spontaneous natural recovery, while late surgery may fail due to irreversible neuromuscular atrophy resulting in suboptimal outcomes.^7^ Therefore, determining appropriate surgical candidates and surgical timing remains a major challenge and is foundational in maximizing outcomes. Moreover, it remains a challenge to determine the inclusion of cervical SCI patients in acute clinical trials. At present, the difficulty in predicting recovery patterns after SCI substantially compounds this inherently difficult decision-making process.

The leading measure used to stratify SCI is the American Spinal Injury Association (ASIA) Impairment Scale (AIS), which has been associated with clinical outcomes following SCI.^8^ However, this relatively simple scale does not have the ability to predict complex patterns of neurological recovery following SCI.^9^ To date, there have been limited efforts to use prospectively collected clinical data to predict long-term neurological outcomes following SCI, including prediction of ambulation,^10^ upper extremity motor score,^11^ and AIS conversions to better grade.^12^ However, currently no prognostic model exists that reliably predicts upper limb functional outcomes following cervical SCI, a critical measure for clinical decision-making. To address this important evidence gap, we developed a clinical prediction tool to prognosticate the likelihood of dependency in activities of daily living (ADLs) requiring upper limb function following cervical SCI.

## Methods

### Study Design and Participants

This prognostic study included a longitudinal cohort of patients with traumatic SCI in the Spinal Cord Injury Model Systems (SCIMS) database in the US.^13^ The SCIMS database represents 6% to 13% of the SCI population in the US and has been described in detail previously.^13^ This study was approved by institutional review board at Washington University, and informed consent was waived because the study included deidentified participant data from the SCIMS database. Data analysis was conducted from September 2021 to June 2022. This study was reported in accordance with Transparent Reporting of a Multivariable Prediction Model for Individual Prognosis or Diagnosis (TRIPOD) reporting guideline checklist.

Neurological examinations were performed at the time of enrollment following SCI and 1-year follow-up. All neurological examinations were performed according to the International Standards for Neurological Classification of Spinal Cord Injury (ISNCSCI) and included motor and sensory scores from each spinal segment. Neurological level of injury and injury severity were defined by the AIS algorithm.^14^

We included patients with cervical SCI aged 15 years and older enrolled in a site participating in SCIMS between 2011 to 2016 with a neurological level of injury between C1 and C8 and an AIS grade between A and D. To predict the outcome in subacute stages of cervical SCI, only patients with baseline neurological examinations (within 30 days of SCI) and complete functional independence measures (FIM) at 1-year follow-up were included. Patients presenting over 30 days after SCI, with AIS grade E or paraplegia, or with missing neurological data and functional outcomes, were excluded.

### Candidate Predictors

We identified candidate clinical and neurological variables potentially associated with outcomes following SCI based on existing studies.^15,16^ Following SCI, older patients have less potential for functional recovery than younger patients.^17^ Surgical decompression following SCI has been significantly associated with improved outcomes.^18^ Additional variables considered were sex, symmetry of SCI, and traumatic etiology.^19,20^ For sensitivity analysis, traumatic etiology was categorized as accidents (including all traumatic mechanisms, such as motor vehicle crashes, assault, and sports-related injuries) and falls (elderly fall-related injuries). To maintain the simplicity of model use, age was categorized as under 60 years and 60 years or older, and surgical decompression was treated as a binary variable (ie, yes/no). As comorbid traumatic brain injury (TBI) has been associated with worse functional outcomes after SCI, we included the presence and absence of TBI in our model and assessed model performance.^21^ The TBI variable was treated as a binary diagnosis: no TBI (including improbable, possible, and mild TBI) and TBI (including moderate and severe TBI).^22^

We chose a battery of neurological measures as assessed by the ISNCSCI.^14^ Motor grade and intact sensations (ie, light touch and pinprick) have been significantly associated with motor recovery following SCI.^23,24,25^ Therefore, neurological variables evaluated were motor scores at C5 to C8 myotomes (manual motor testing grades 0 through 5) and light touch score (LTS) and pinprick score (PPS) (with 0 representing absent; 1, impaired; and 2, normal) at C2 through C8 dermatomes.^24^ Significant correlation exists between LTS and PPS,^26^ and the reliability of LTS is known to be higher than PPS due to the complexity of discriminating sharp from dull sensations.^27^ Therefore, we initially included only LTS to simplify clinical use of the model. At each spinal level, only the best-performing motor and sensory scores (ie, from right or left upper limb) were included in the analysis. Finally, severity of SCI was defined by AIS grading.^14^ Symmetrical SCI was defined as right and left motor levels being the same and asymmetrical defined as 1 level difference or more.^20^ To develop the prognostic model for early prediction of outcome (within the subacute phase of SCI), we included patients with baseline neurological examinations performed within 30 days of SCI.

### Study Outcome

The primary outcome was the composite of dependency on major ADLs assessed by the FIM 1 year after SCI. The FIM is a validated measure employed in various large SCI data sets in North America,^28,29,30^ enabling the harmonization of data and providing opportunity for external validation of our model in future. The FIM voluntary motor domains have been shown to correlate with neurological function and reflect functional status by varying severity of SCI.^31,32^ Each domain ranges from a score of 1 (total assistance) to 7 (complete independence). Patients with a score of at least 5 can perform the function on their own, while patients scoring 4 or below require minimal to full helper assistance in performing the function.^32^ Since our goal was to accurately identify those patients who have severe functional dependence, we chose a threshold of 4 and below for defining dependency in each domain.^32^ Because we aimed to identify patients who had severe upper limb impairment requiring assistance in a range of ADLs, we chose a composite outcome of being dependent on at least 3 FIM domains including eating, bladder management, transfers (ie, to bed, chair, or wheelchair), and locomotion. These functions were chosen as they have been known to be the most relevant for tetraplegic patients that can be restored by upper limb surgery.^20,33^

### Statistical Analysis

Frequencies and proportions for categorical variables and mean averages for continuous variables were estimated. To develop a prediction model, we included all candidate predictors in multivariable logistic regression models. To select a parsimonious model, an exhaustive search was performed with a maximum of 7 predictors in various combinations and all models were evaluated (eMethods in Supplement 1).^34^ The final model was chosen based on the lowest Akaike information criteria (AIC), the smallest number of predictors, and greater ease of clinical use.

Data were split into derivation (2011-2014) and temporal validation (2015-2016) cohorts. The model performance in terms of discriminatory accuracy was quantified by *C* statistic and calibration was evaluated. A *C* statistic value of 0.50 represented discrimination no better than chance and 1.00 represented perfect discrimination. Model calibration, which is the agreement between predicted and observed outcome, was assessed by a calibration plot, calibration slope, and intercept. Internal validation to correct optimism (ie, overfitting) was performed by bootstrapping 1000-samples and 10-fold cross-validation of derivation data. Optimism-corrected *C* statistics and corresponding 95% CIs were obtained.

A clinically usable scoring system was developed by assigning integer points to each variable based on relative regression coefficients in the final model.^35^ Cut-offs of scores at various predicted probability thresholds were determined at values with maximum sensitivity, specificity, positive predictive value, and negative predictive value. The accuracy of the newly developed score was compared with the AIS grading. Moreover, temporal validation was performed by applying the prediction model and the scoring algorithm to the temporal validation cohort.

Several sensitivity analyses were performed. First, improvement of predictive capacity of the model was assessed by adding PPS. Second, the utility of the model by using neurological examinations within the first 15 days vs 15 to 30 days of SCI was assessed. Third, predictive performance of model was evaluated separately in patients with high tetraplegia (C1-C4) and low tetraplegia (C5-C8). Fourth, the performance of our model was compared with AIS grading, which has been considered as the most important predictor of SCI outcomes.^36,37^ Finally, the association of TBI with predictive performance of the model was assessed by testing TBI in the model and evaluating model performance separately in patients with and without TBI. In addition, the association of traumatic etiology with performance of our model was also evaluated. The threshold for significance was set at 2-tailed α < .05. All analyses were performed in R version 4.2.1 (R Project for Statistical Computing). Exhaustive model search was performed using glmuti version 1.0.8.^34^

## Results

### Cohort Characteristics

**Figure 1.**
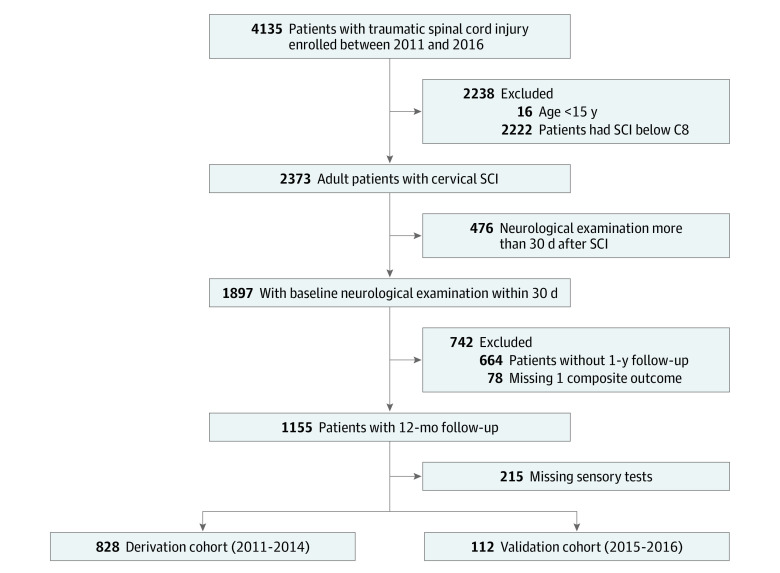
Flowchart of Cohort Selection

Between 2011 and 2016, 4135 patients with traumatic SCI were enrolled in a site participating in SCIMS, and 2373 patients had cervical SCI (C1-C8) at the time of admission. Of the patients with cervical SCI, 1897 patients had a neurological examination within 30 days of SCI. The functional independence measure (FIM) was available in 1155 patients at 1-year follow-up, with 940 having complete initial neurological examinations (Figure 1). Overall, 118 of these 940 patients (13%) were dependent at 1-year. Among dependent patients, 92 (78%) were men, and 83 (70%) were younger than 60 years (Table 1). Seventy-three dependent patients (62%) experienced AIS grade A SCI, and motor vehicle accidents were the most common cause. The clinical characteristics of patients in the derivation cohort were similar to those in the validation cohort (Table 1). The clinical characteristics of excluded patients were similar to the included patients (eTable 1 in Supplement 1).

**Table 1.** Clinical Characteristics of Patients in Derivation and Temporal Validation Cohorts

Demographics	Derivation cohort patients, No. (%) (n = 828)		*P* value^a^	Temporal validation cohort patients, No. (%) (n = 112)		*P* value^a^
	No outcome (n = 710)	Outcome (n = 118)		No outcome (n = 97)	Outcome (n = 15)	
Age						
<60 y	542 (76)	83 (70)	.20	67 (69)	11 (73)	.81
≥60 y	168 (24)	35 (30)		30 (31)	4 (27)	
Sex						
Men	577 (81)	92 (78)	.47	73 (75)	11 (73)	.90
Women	133 (19)	26 (22)		24 (25)	4 (27)	
Traumatic etiology						
Motor vehicle	272 (38)	49 (41)	.001	43 (44)	5 (33)	.04
Assault	38 (5)	17 (14)		2 (2)	2 (13)	
Sports injury	119 (17)	8 (7)		10 (10)	2 (13)	
Fall	272 (38)	43 (36)		42 (43)	5 (33)	
Other	4 (1)	1 (1)		0	0	
Iatrogenic	5 (1)	0		0	1 (7)	
Time of neurological examination following SCI						
<15 d	473 (67)	47 (40)	<.001	72 (74)	5 (33)	.005
≥15 d	237 (33)	71 (60)		25 (26)	10 (67)	
AIS grade						
A	158 (22)	73 (62)	<.001	15 (15)	6 (40)	.005
B	101 (14)	21 (18)		13 (13)	4 (27)	
C	170 (24)	20 (17)		25 (26)	4 (27)	
D	281 (40)	4 (3)		44 (45)	1 (7)	
Symmetry of SCI on both sides						
Symmetrical	383 (54)	86 (73)	<.001	54 (56)	8 (53)	.91
Asymmetrical	327 (46)	32 (27)		43 (44)	7 (47)	

Abbreviations: AIS, American Spinal Injury Association impairment scale; SCI, spinal cord injury.

^a^ Comparisons were made using χ^2^ or Fishers exact test as appropriate.

### Prediction Model

In the derivation cohort (828 patients), dependency on ADL at 1-year follow-up (ie, the primary outcome) was present in 118 patients (14%). Age, sex, upper limb motor scores, upper limb light touch scores, and several other neurological measures were identified as important predictor variables in all models explored by exhaustive search (eFigure 1 in Supplement 1). Following model search, 10 models with equivalent performances of predictor variables in various combinations were evaluated (eTable 2 in Supplement 1). The final selected model included 6 predictors—age (age 60 years or older: OR, 2.31; 95% CI, 1.26-4.19), gender (men: OR, 0.60; 95% CI, 0.31-1.17), C5 LTS (OR, 0.44; 95% CI, 0.44-1.01), C8 LTS (OR, 036; 95% CI, 0.24-0.53), C5 motor score (OR, 0.74; 95% CI, 0.60-0.89), and C6 motor score (OR, 0.61; 95% CI, 0.49-0.75) (Table 2). The final model had a *C* statistic of 0.91 (95% CI, 0.88-0.95).

**Table 2.** Final Model Predicting the Dependency in ADLs 1-Year After Trauma Cervical SCI^a^

Predictors	β coefficient	Odds ratio (95% CI)
Age		
<60 y	1 [Reference]	1 [Reference]
≥60 y	0.83	2.31 (1.26 to 4.19)
Sex		
Women	1 [Reference]	1 [Reference]
Male	–0.50	0.60 (0.31 to 1.17)
Light touch score		
C5	–0.40	0.67 (0.44 to 1.01)
C8	–1.02	0.36 (0.24 to 0.53)
Motor score		
C5	–0.30	0.74 (0.60 to 0.89)
C6	–0.49	0.61 (0.49 to 0.75)

Abbreviations: ADLs, activities of daily living; SCI, spinal cord injury.

^a^ Model intercept was 1.66.

**Figure 2.**
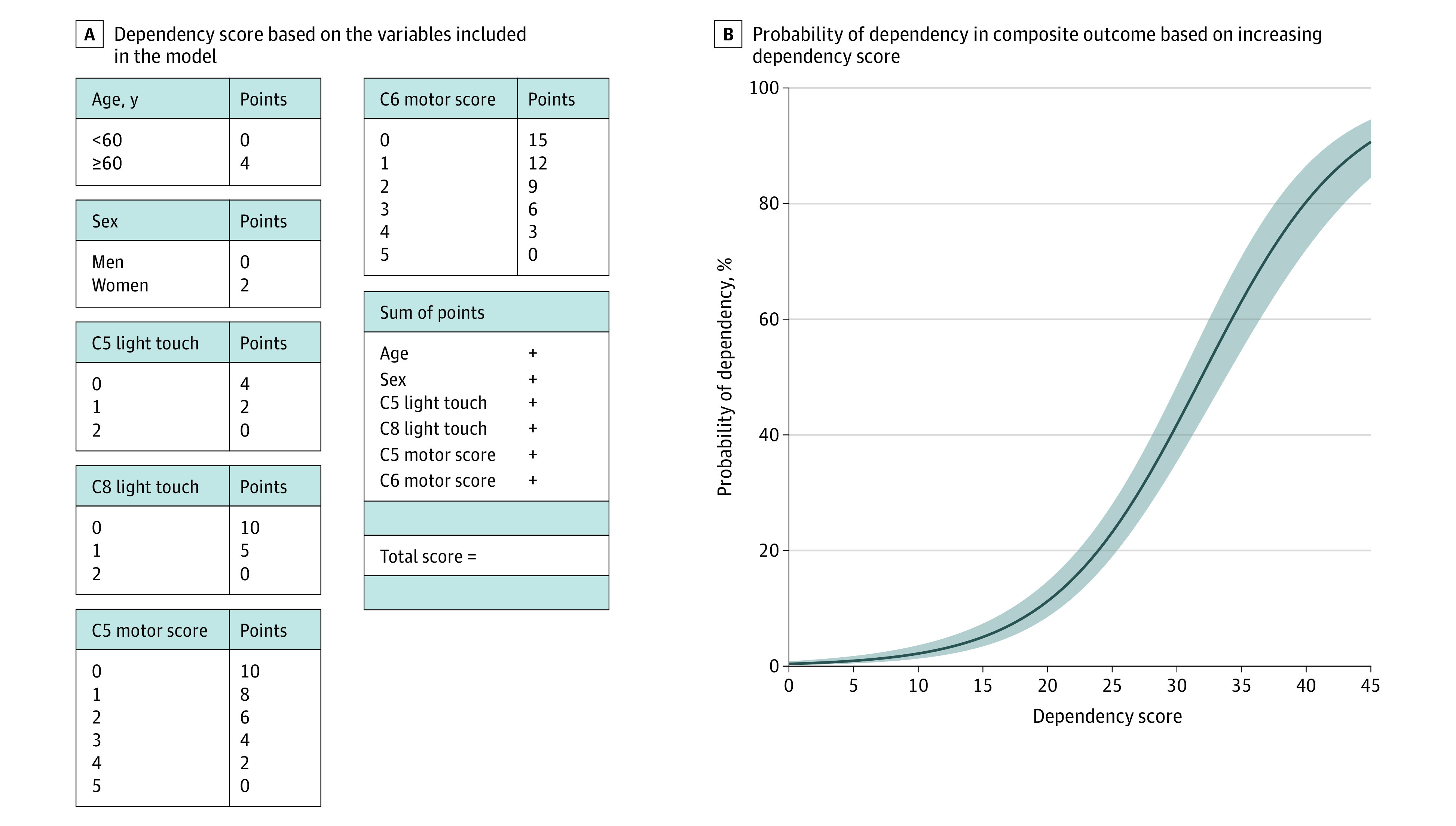
Model Dependency Score Variables and Probability of Dependency The shaded area represents 95% confidence interval based on regression of the prediction rule.

Although a model with 7 predictors (the 6 predictors above and spine surgery) had the lowest AIC, considering the same *C* statistic (0.91; 95% CI, 0.88-0.95) and ease of clinical use, we selected the parsimonious model as the final model. Based on the final 6-predictor model, an integer-based dependency score was developed (Figure 2). The dependency score ranged from 0 to 45 points with higher scores associated with increasing probability of dependency in ADLs (eFigure 2 in Supplement 1).

### Model Performance and Validation

**Figure 3.**
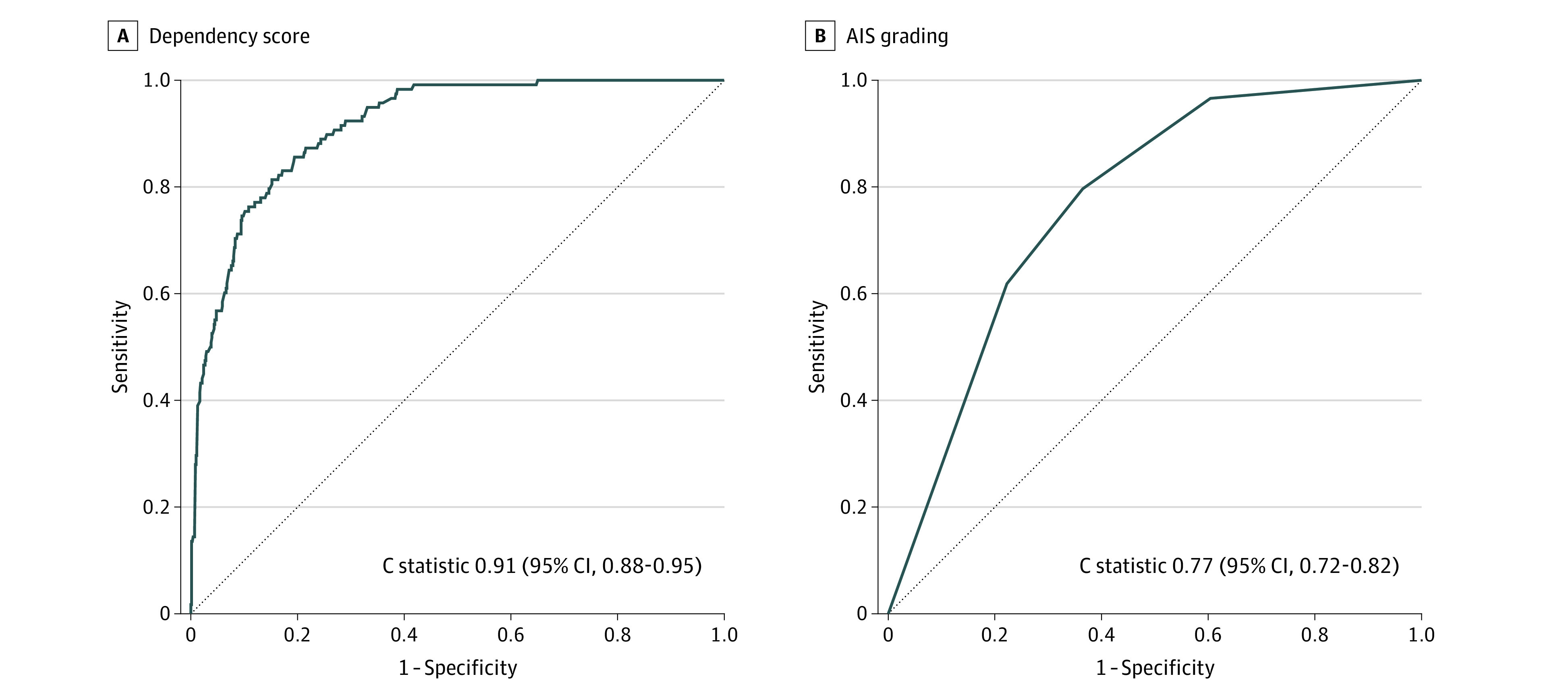
Comparison of Performance of Dependency Score After SCI vs AIS Grading in Predicting Dependency in ADLs at 1-Year After SCI ADLs indicates activities of daily living; AIS, American Spinal Cord Injury Association impairment scale; AUC, area under the operating curve. The improvement in AUC after using prediction model as compared to the AIS grading was 0.14 (95% CI, 0.10-0.18, *P* < .001).

The 6-predictor model showed excellent ability to discriminate between patients with and without dependency on ADLs at 1-year follow-up (optimism-corrected *C* statistic, 0.90; 95% CI, 0.88-0.93). The agreement between observed and predicted outcome also showed good calibration (eFigure 3 in Supplement 1). Adding C8 PPS scores, C7 motor scores, level of tetraplegia (high [C1-C4] vs low [C5-C8]), presence of TBI (no-to-mild TBI vs moderate-severe TBI), and timing of neurological examination (first 15 days vs 15 to 30 days of SCI) to the 6-predictor model did not significantly improve the discrimination of the model (*P* = .37, .27, .90, .39, and .42 respectively). In addition, the model’s discriminatory ability was good regardless of timing of neurological examination (first 15 days vs 15 to 30 days of SCI), level of tetraplegia (high [C1-C4] vs low [C5-C8]), presence of TBI (no-to-mild TBI vs moderate-severe TBI), and traumatic etiology (accidents vs falls) (eTable 3 in Supplement 1). The predictive performance of the model was maintained in each of the AIS grades (eFigure 4 in Supplement 1). The dependency score significantly outperformed the AIS grading in predicting the outcome. The improvement in *C* statistic was 0.14 (95% CI, 0.10-0.18; *P* < .001) (Figure 3).

In the validation cohort (112 patients), dependency on ADL at 1-year follow-up occurred for 15 patients (13%). When the 6-predictor model was applied to the validation cohort, the *C* statistic was 0.89 (95% CI, 0.78-0.99). Despite the smaller sample size in the validation cohort, the model maintained good calibration (eFigure 5 in Supplement 1). The dependency score also outperformed the AIS grading in temporal-validation cohort (improvement in *C* statistic, 0.15; 95% CI, 0.02-0.27; *P* = .02).

### Clinical Performance of Prediction Model

The potential clinical performance of applying the dependency score in the SCI patients being evaluated within 30 days of SCI to plan for reconstructive therapies was also assessed (eTable 4 in Supplement 1). At a lower predicted probability threshold of 5% or higher with a score of 14 or greater, 42% of patients were stratified in the dependent category with high sensitivity of 94% (95% CI, 88%-98%). While at a higher predicted probability threshold of 50% or higher with a score of 31 or greater, 11% of patients were stratified in the dependent category with high specificity of 96% (95% CI, 94%-97%). Negative predictive values were 89% or higher for each of the probability thresholds.

## Discussion

In this prognostic study, we developed a novel prognostic score that can be used to predict upper limb functional outcomes in patients with traumatic cervical spinal cord injury during the subacute phase. We used a large prospective database to develop a multivariable model based on age, sex, and 4 neurological tests, which predicted long-term dependency requiring upper limb function with reasonable accuracy. The prediction model was well-calibrated and had high discrimination, effectively identifying patients who would eventually need reconstructive or rehabilitative interventions to reanimate upper limb function. In addition, we showed that this model had superior prognostic performance compared with the AIS grading in predicting dependency in tetraplegic patients. We provide a statistically robust model which is easy to use in clinical settings.

Various prediction models have been developed to prognosticate functional recovery following traumatic cervical SCI.^11,38,39,40,41,42^ These models used acute clinical examination,^25,41^ MRI metrics,^39,42^ functional tests (eg, grip patterns),^38^ and electrophysiological measures^40^ to predict functional outcomes after SCI. Although these models have demonstrated high predictive performances, they were limited by relatively small sample sizes and utilized metrics, such as neurophysiological tests and grasp testing, which might be cumbersome for clinicians and impractical for broad clinical application. For tetraplegic patients, upper limb motor ability is considered the most important for functional independence, as it best enables patients to replace lost bodily functions (eg, driving a wheelchair to replace lost ambulation).^3,4^ However, there remains a need for a rigorously derived prediction model that quantifies the long-term probability of upper limb function following tetraplegia.

Following cervical SCI, reconstructive surgery, such as nerve and tendon transfers, remains the most reliable options to reanimate lost upper limb function.^5,43^ There is a narrow window of opportunity for the timing of these surgeries, as permanent denervation and atrophy occurs in the muscles involved in the injured segment of the spinal cord.^7,44^ However, it is important to consider that following tetraplegia, patients have significant potential for spontaneous recovery in the early phase of SCI.^15,45^ Therefore, early surgery might obliterate the spontaneous natural recovery following SCI.^7^ This heterogeneity of clinical presentation complicates clinical decision-making regarding the appropriateness and timing of surgical intervention. This newly developed score after SCI may serve as a clinical decision support tool preventing unnecessary resource investment and irreversible reconstructive surgeries in patients with high probability of functional improvement. Our goal was to use simple neurological examinations familiar to most clinicians for prediction of long-term functional dependency during early (subacute) phases of SCI. This information can be used to ascertain the appropriate referral to the neurosurgery clinics and for patient counselling.

Prior studies have demonstrated that early after SCI postinjury edema and hemorrhage results in spinal shock that confounds the reliability of early (ie, within 72 hours after SCI) neurological examinations.^46,47^ Therefore, when predicting the long-term functional outcomes, delayed neurological examinations may have better predictive capacity.^16,47^ However, in our sensitivity analysis, we found no significant difference in the performance of the prediction model either by adding the timing of neurological examination variable in the final model or by subgroup analysis in early (first 15 days) vs delayed (15 to 30 days) examination groups. In addition, we assessed the utility of the prognostic model in high tetraplegia (C1-C4) vs low tetraplegia (C5-C8). The model performed well in both high and low tetraplegia subgroups. Concurrent traumatic brain injury can significantly affect the outcomes of spinal cord injury.^21^ In our sensitivity analysis, our model had high discrimination in patients with and without TBI; however, it had moderately lower calibration in patients with moderate-severe TBI, likely reflecting mild heterogeneity in recovery of SCI patients with comorbid TBI.^21^ Finally, the traumatic etiology of SCI may significantly affect outcomes.^19^ However, our model performed well in both accidental and fall-related SCIs. Overall, our results suggest that this prognostic model retained its performance regardless of the timing of neurological examination, level of cervical SCI, presence of concurrent TBI, and traumatic etiology, and therefore could be applied in subacute clinical settings.

Although AIS grading was designed to track SCI recovery, multiple studies have evaluated its prognostic value for sensorimotor and functional outcomes.^36,37^ Traditionally, AIS grading has been considered as one of the most important predictors for prognosticating recovery following SCI.^9,48^ However, the calculation of AIS grades requires comprehensive ISNCSCI scoring algorithm, which is time consuming and not feasible in the clinical settings. In comparative analyses, our prediction rule outperformed the AIS grading with significant improvement in discrimination.

Our prediction model can be applied to predict dependency in a composite of FIM items including eating, bladder management, transfers, and locomotion. These functions were chosen because they represent the most relevant activities reflecting upper extremity function that can be restored with upper limb surgery in tetraplegic patients.^4,20,33^ We chose a higher cutoff of dependency in 3 or more functions for our composite outcome to identify severely dependent patients in a range of ADLs. These patients would have the greatest potential of benefiting from early reconstructive surgery.

Recent epidemiological data suggests that every year approximately 9000 patients suffer from cervical SCI resulting in 148 000 patients living with tetraplegia in the US.^1,2^ Yet, upper extremity reconstructive surgery remains underutilized; based on one estimate, only 14% of the eligible SCI population in the US undergoes these procedures.^49^ The major reasons for such low rates of surgical utilization are the lack of either a patient’s or clinician’s knowledge about treatment and clinical uncertainty in prognosis of functional outcomes following tetraplegia.^49,50^ With early prediction of upper limb function, our results could inform clinical practice guidelines to provide stronger recommendations supporting the role of reconstructive surgery following cervical SCI. Additionally, this prediction tool may be used to identify dependent tetraplegic patients for early enrollment in acute interventional clinical trials.

### Limitations

Our study has important strengths, including a large, multicenter cohort, temporal validation, and rigorous statistical methods. Nonetheless, it also has several limitations. First, because acute neurological examinations were not available, the utility of this prediction model in hyperacute phases (within 24 hours) of SCI is not known. Future work should investigate the prognostic capability of this score during ultra-early phase of SCI. Second, although our prediction score was developed using a large patient cohort and performed well in temporal validation cohort, our model has not been externally validated. Future external validation is needed to verify the generalizability of our findings. Third, although we developed an easy-to-use scoring algorithm, physician acceptance and implementation of the score in practice have not been evaluated. Finally, the SCIMS data set used in model development had only functional independence measures available. However, recent SCI studies adopted the spinal cord independence measure and spinal cord injury functional index.^51,52^ Future work should validate this tool in these domains.

## Conclusions

In this prognostic study, a simple multivariable model using early neurological examinations was able to predict upper limb functional outcomes 1-year following cervical spinal cord injury. This tool can assist clinical decision-making for early reconstructive surgeries to reanimate upper limb function in patients with tetraplegia.

## Notes

Supplement. **eMethods.** Model Selection Strategy with Final Set of Predictor Variables **eTable 1.** Clinical Characteristics of Included and Excluded Patients **eTable 2.** The 10 Best Models Evaluated After Exhaustive Model Search **eTable 3.** Performance of Prediction Model in Stratified Derivation Cohort **eTable 4.** Performance of Prediction Rule in Varying Thresholds of Predicted Probability and Points of Score in Predicting Composite Outcome in Dependency **eFigure 1.** Fitted Models and Final Predictor Variables **eFigure 2.** Distribution of Dependency Score in Derivation Cohort **eFigure 3.** Optimism Corrected Calibration Plot After Internal Validation **eFigure 4.** The SCI Prognostic Score Dichotomized by Each AIS Grade **eFigure 5.** Calibration Plot in Temporal Validation Cohort

Supplement 2.
Data Sharing Statement
