# Supplementary material for: Derivation and Validation of a Clinical Prediction Rule for Upper Limb Functional Outcomes After Traumatic Cervical Spinal Cord Injury
Source: JAMA Netw Open. 2022 Dec 21;5(12):e2247949. doi: 10.1001/jamanetworkopen.2022.47949 (PMC9857030; doi:10.1001/jamanetworkopen.2022.47949)
Supplement: Supplement. — eMethods. Model Selection Strategy with Final Set of Predictor Variables eTable 1. Clinical Characteristics of Included and Excluded Patients eTable 2. The 10 Best Models Evaluated After Exhaustive Model Search eTable 3. Performance of Prediction Model in Stratified Derivation Cohort eTable 4. Performance of Prediction Rule in Varying Thresholds of Predicted Probability and Points of Score in Predicting Composite Outcome in Dependency eFigure 1. Fitted Models and Final Predictor Variables eFigure 2. Distribution of Dependency Score in Derivation Cohort eFigure 3. Optimism Corrected Calibration Plot After Internal Validation eFigure 4. The SCI Prognostic Score Dichotomized by Each AIS Grade eFigure 5. Calibration Plot in Temporal Validation Cohort [file jamanetwopen-e2247949-s001.pdf]

Supplemental Online Content

Javeed S, Greenberg JK, Zhang JK, et al. Derivation and validation of a clinical prediction rule for upper limb functional outcomes after traumatic cervical spinal cord injury. *JAMA Netw Open.* 2022;5(12):e2247949.
doi:10.1001/jamanetworkopen.2022.47949

eMethods. Model Selection Strategy with Final Set of Predictor Variables

eTable 1. Clinical Characteristics of Included and Excluded Patients

eTable 2. The 10 Best Models Evaluated After Exhaustive Model Search

eTable 3. Performance of Prediction Model in Stratified Derivation Cohort

eTable 4. Performance of Prediction Rule in Varying Thresholds of Predicted Probability and Points of Score in Predicting Composite Outcome in Dependency

eFigure 1. Fitted Models and Final Predictor Variables

eFigure 2. Distribution of Dependency Score in Derivation Cohort

eFigure 3. Optimism Corrected Calibration Plot After Internal Validation

eFigure 4. The SCI Prognostic Score Dichotomized by Each AIS Grade

eFigure 5. Calibration Plot in Temporal Validation Cohort

This supplemental material has been provided by the authors to give readers additional information about their work.

© 2022 Javeed S et al. *JAMA Network Open.*

eMethods. Model Selection Strategy with Final Set of Predictor Variables

Automated exhaustive model search was performed using the “glmulti” package in R. All candidate predictor variables were included in the initial model. Candidate predictor variables included age, sex, symmetrical SCI, spine surgery, light touch scores from C2 to C8 dermatomes, and motor scores from C5 to C8 myotomes. Because the aim of this study was to develop a simple prognostic model with minimal patient discomfort and time efficient for physicians, we did not include the aggregate upper extremity motor scores and AIS grading into the model. As shown in eFigure 1, 16,950 possible models were fitted with candidate predictor variables in various combinations with maximum of seven predictor variables in each model to avoid overfit and retain simplicity of clinical use. The importance of each predictor variable (i.e., summed weight of each variable appearing in all models fitted) was quantified. The best models were selected based on lowest Akaike Information Criteria (AIC) given in eTable 2. The final model was chosen with lowest number of predictor variables and ease of clinical use.

© 2022 Javeed S et al. *JAMA Network Open.*

eTable 1: Clinical Characteristics of Included and Excluded Patients in Derivation and Temporal Validation Cohorts

	No (%) of Patients			
	Derivation Cohort		Temporal Validation Cohort	
	Included (n=828)	Excluded (n=412)	Included (n=112)	Excluded (n=524)
**Demographics**				
**Age**				
<60	625 (75)	288 (70)	78 (70)	375 (72)
≥60	203 (24)	124 (30)	34 (30)	149 (28)
**Sex**				
Male	669 (81)	341 (83)	84 (75)	402 (77)
Female	159 (19)	71 (17)	28 (25)	122 (23)
**Traumatic Etiology**				
Motor Vehicle Accident	321 (39)	140 (35)	48 (43)	190 (38)
Assault	55 (7)	28 (7)	4 (3)	31 (6)
Sports injury	127 (15)	44 (11)	12 (11)	62 (12)
Fall	315 (38)	177 (44)	47 (42)	214 (43)
Other	5 (0.6)	18 (4)	0 (0)	22 (4)
Iatrogenic	5 (0.6)	5 (1)	1 (0.8)	5 (1)
**Neurological examination following SCI**				
Missing motor scores	–	0 (0)	–	1 (0)
Missing sensory scores	–	88 (21)	–	6 (1)
Neurological examination >30 days	–	99 (24)	–	102 (19)
**Missing functional measures at one year**				
Eating	–	412 (100)	–	524 (100)
Bladder management	–	410 (99)	–	524 (100)
Transfers	–	412 (100)	–	524 (100)
Locomotion	–	411 (99)	–	524 (100)
**Severity of SCI**				
AIS Grade A	231 (28)	126 (31)	21 (19)	133 (26)
AIS Grade B	122 (15)	65 (16)	17 (15)	63 (12)
AIS Grade C	190 (23)	83 (20)	29 (26)	115 (22)
AIS Grade D	285 (34)	134 (33)	45 (40)	207 (40)
**Symmetry of SCI on both sides**				
Symmetrical	469 (57)	237 (57)	62 (55)	299 (57)
Asymmetrical	359 (43)	175 (43)	50 (45)	225 (43)

AIS: American spinal injury association; SCI: Spinal cord injury.

© 2022 Javeed S et al. *JAMA Network Open.*

eTable 2. The 10 Best Models Evaluated After Exhaustive Model Search With Maximum of 7 Predictors				
**Model**	**Predictor Variables**	**AIC**	**Weights[b]**	**AUC (95% CI)**
1	Age, Sex, Spine surgery, C5 LTS, C8 LTS, C5 MS, C6 MS	274.99	0.047	0.91 (0.88-0.95)
**[a]2**	**Age, Sex, C5 LTS, C8 LTS, C5 MS, C6 MS**	**275.08**	**0.045**	**0.91 (0.88-0.95)**
3	Age, Sex, C4 LTS, C5 LTS, C8 LTS, C5 MS, C6 MS	275.19	0.043	0.91 (0.88-0.95)
4	Age, Sex, C5 LTS, C8 LTS, C5 MS, C6 MS, C8 MS	275.83	0.031	0.92 (0.89-0.95)
5	Age, Sex, Spine surgery, C5 LTS, C8 LTS, C5 MS, C7 MS	276.04	0.028	0.91 (0.88-0.95)
6	Age, Sex, C5 LTS, C8 LTS, C5 MS, C6 MS, C7 MS	276.05	0.028	0.91 (0.88-0.95)
7	Age, Sex, C5 LTS, C6 LTS, C8 LTS, C5 MS, C6 MS	276.24	0.025	0.91 (0.88-0.95)
8	Age, Sex, C4 LTS, C5 LTS, C8 LTS, C5 MS, C7 MS	276.43	0.023	0.91 (0.88-0.95)
9	Age, Sex, C5 LTS, C8 LTS, C5 MS, C7 MS	276.53	0.022	0.91 (0.88-0.95)
10	Age, Spine surgery, C4 LTS, C5 LTS, C8 LTS, C5 MS, C6 MS	276.95	0.018	0.91 (0.88-0.95)

[a] Final model was selected based on ease of clinical use and lowest number of predictor variables.
[b] Model weights (also called "Akaike weights") for a particular model can be regarded as the probability that the model is the best model out of all the models considered/fitted.

Abbreviations: AIC: Akaike information criteria; AUC: area under curve; LTS: light touch score; MS: motor score.

© 2022 Javeed S et al. *JAMA Network Open.*

eTable 3: Performance of the prediction model in the derivation cohort, stratified by timing of neurological examination, level of cervical SCI, traumatic brain injury, and mechanism of traumatic SCI				
**Strata**	**Dependency in ADLs, n (%)**	**Calibration intercept**	**Calibration slope**	**C Statistic (95% CI)**
**Timing of baseline neurological examination**				
<15 days of SCI (n=520)	47 (9)	-0.1	0.90	0.90 (0.84-0.96)
15-30 days of SCI (n=308)	71 (23)	-0.01	0.93	0.92 (0.87-0.96)
**Level of cervical SCI[a]**				
High C1-C4 (n=536)	110 (20)	-0.07	0.92	0.88 (0.84-0.93)
Low C5-C8 (n=292)	8 (3)	-0.52	0.77	0.95 (0.85-0.99)
**Presence of co-morbid traumatic brain injury[b]**				
No-Mild TBI (n=643)	79 (12)	-0.04	0.96	0.90 (0.86-0.95)
Moderate-Severe TBI (n=117)	26 (22)	-0.16	0.73	0.94 (0.87-0.99)
**Traumatic Etiology of SCI**				
Accidents[c] (n=503)	74 (15)	-0.05	0.93	0.92 (0.88-0.96)
Falls (n=325)	44 (13)	-0.11	0.90	0.90 (0.84-0.96)

ADL: Activities of daily living; SCI: Spinal cord injury. All performance measures are optimism corrected after 10-fold cross validation.

[a] Level of cervical SCI defined by neurological level of injury.
[b] TBI status was unknown in 68 patients in derivation cohort. Based on TBI Model Systems Severity Scale
[c] Accidents included motor vehicle crash, assault, and sports injuries.

© 2022 Javeed S et al. *JAMA Network Open.*

**eTable 4: Performance of prediction rule in varying thresholds of predicted probability and points of score in predicting composite outcome in dependency**						
**Predicted probability**	**>1%**	**>5%**	**>15%**	**>25%**	**>50%**	**>75%**
Points in score within 30 days of SCI	>6	>14	>21	>23	>31	>37
Patients stratified to increased probability of dependency %	70	42	26	19	11	4
Sensitivity % (95% CI)	99 (95-100)	94 (88-98)	82 (74-89)	74 (65-81)	51 (41-60)	25 (17-33)
Specificity % (95% CI)	35 (32-39)	67 (63-70)	84 (81-86)	90 (87-92)	96 (94-97)	99 (98-100)
Positive predictive value % (95% CI)	20 (17-24)	32 (27-37)	46 (39-53)	54 (46-62)	68 (57-78)	83 (66-93)
Negative predictive value % (95% CI)	100 (98-100)	99 (97-99)	97 (95-98)	95 (93-97)	92 (90-94)	89 (86-91)
95% Confidence Intervals (CI) were calculated by exact method. SCI: Spinal cord injury						

© 2022 Javeed S et al. *JAMA Network Open.*

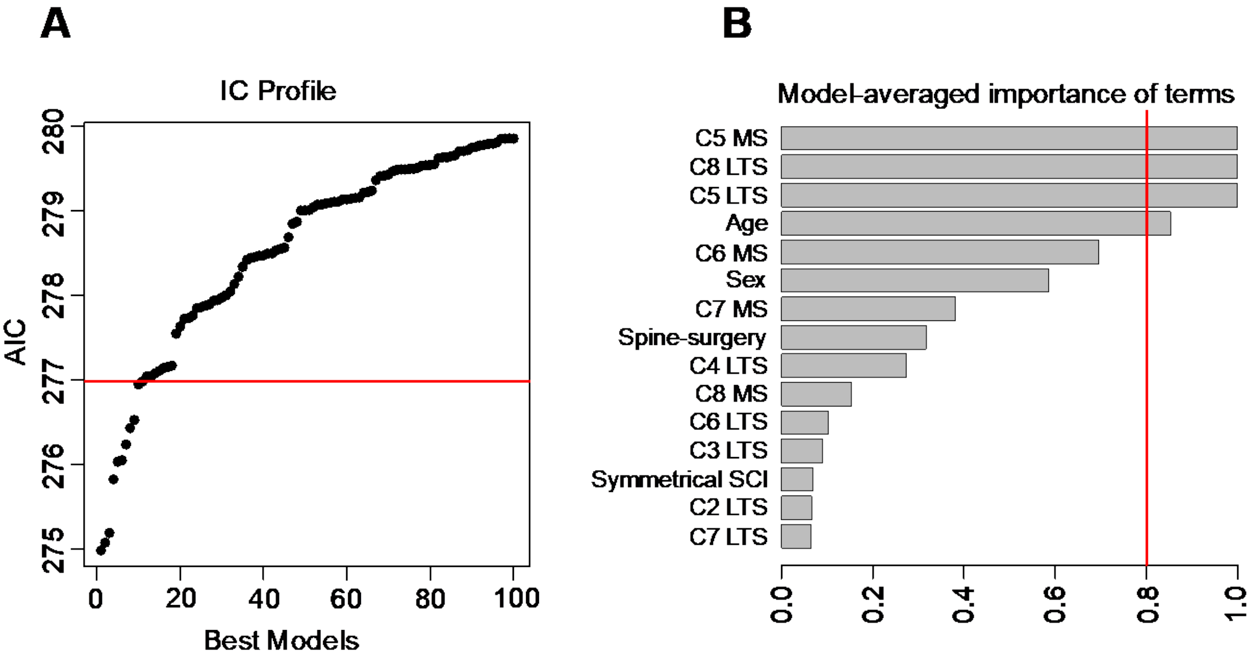

eFigure 1. Fitted Models and Final Predictor Variables.
A) Automated selection of best models fitted, based on Akaike information criteria (AIC). The red line differentiates between models whose AIC value is more than 2 units away from that of the "best" model (i.e., the model with the lowest AIC). **B)** The importance value shows variables selected by most models and have a large weight resulting in higher importance value (calculated as sum of weights/probabilities for the models in which the variable appears). The red line shows a cut-off of 0.8 to differentiate the most important variables. MS: motor score; LTS: light touch score.

© 2022 Javeed S et al. *JAMA Network Open.*

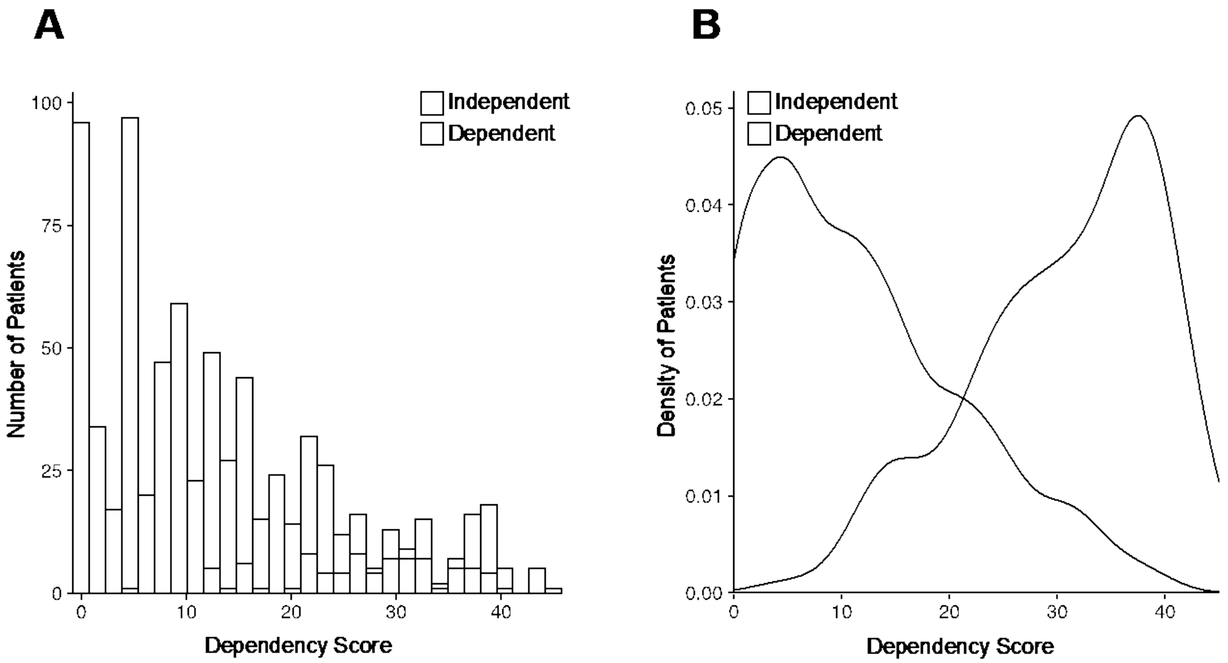

eFigure 2. Distribution of Dependency Score in Derivation Cohort

A) Overall distribution of the prediction score dichotomized by dependency in ADLs (primary outcome). **B)** The density plot showing higher scores in dependent tetraplegic patients as compared to patients who were independent.

© 2022 Javeed S et al. *JAMA Network Open.*

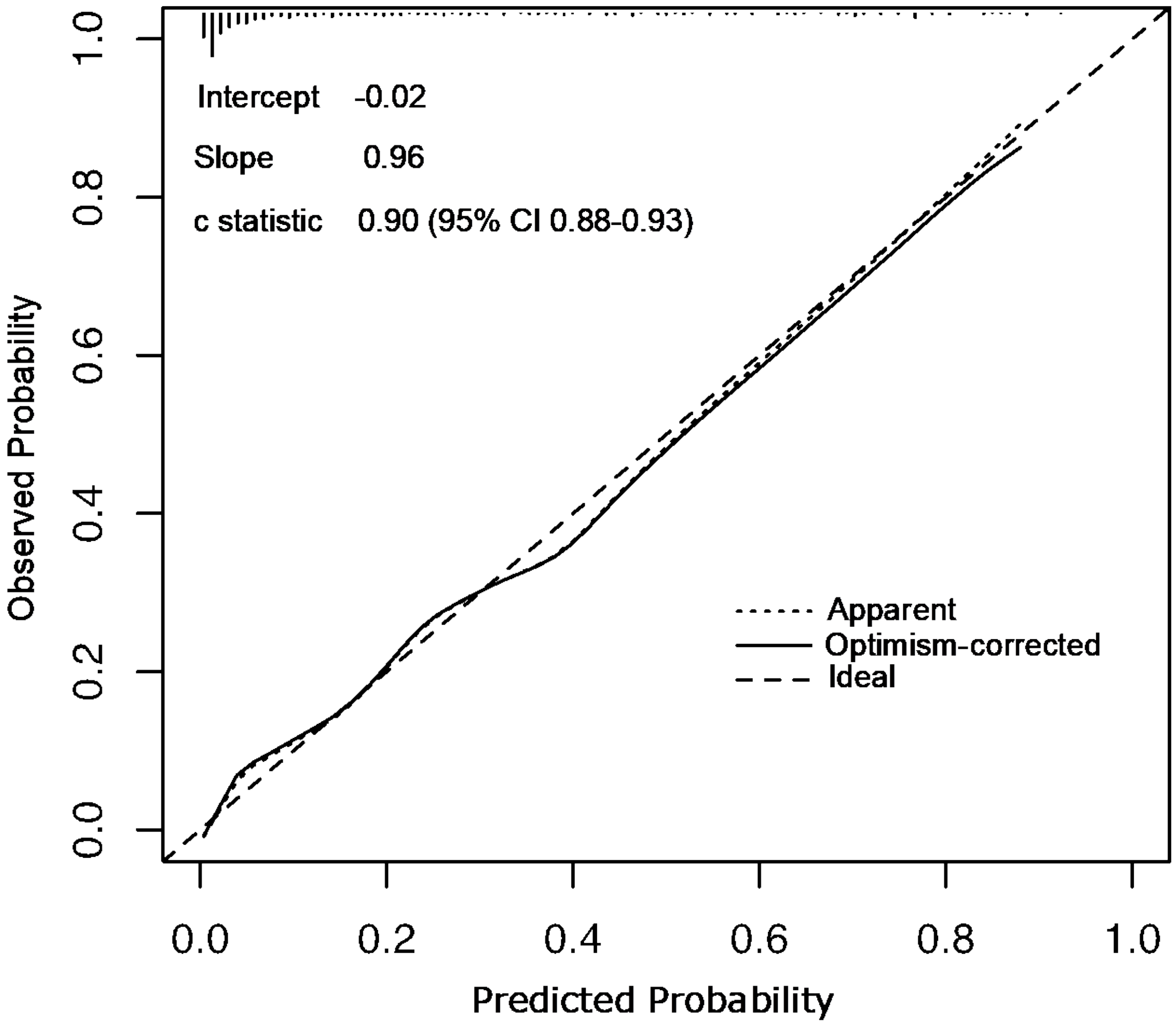

eFigure 3. Optimism Corrected Calibration Plot After Internal Validation
Calibration plot showing observed *vs.* predicted probabilities of dependency in ADLs. Ideal: model with perfect calibration, Apparent: calibration of original model, and optimism-corrected: calibration corrected after internal validation with bootstrapping-1000 samples.

© 2022 Javeed S et al. *JAMA Network Open.*

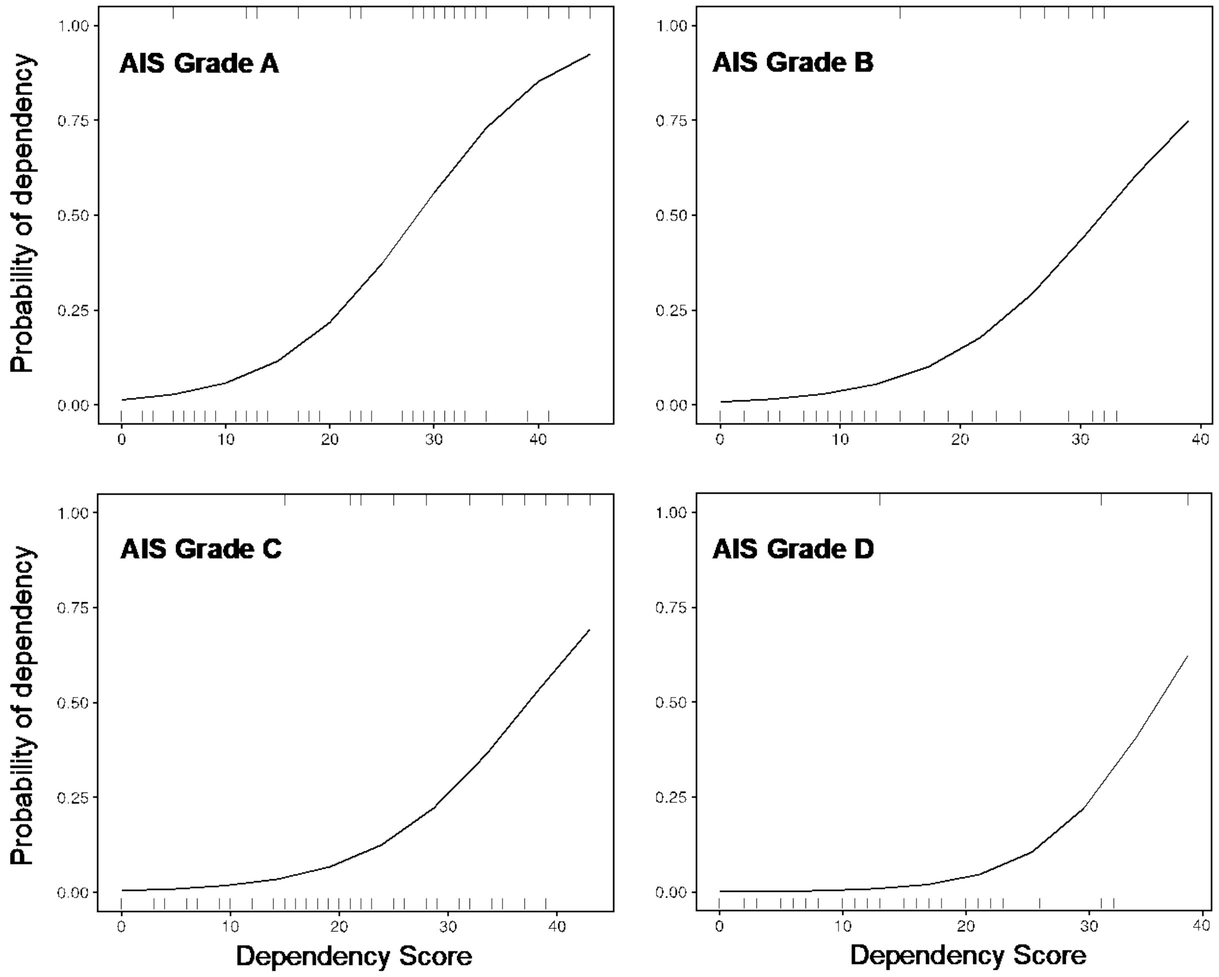

eFigure 4: The SCI Prognostic Score Dichotomized by Each AIS Grade. The shaded area is showing the 95% confidence interval of the regression of the dependency score. Each vertical black line at upper border represents the score of patients who remained dependent in ADLs and at lower border represents score of patients who gained independence in ADLs. AIS: American spinal injury association impairment scale.

© 2022 Javeed S et al. *JAMA Network Open.*

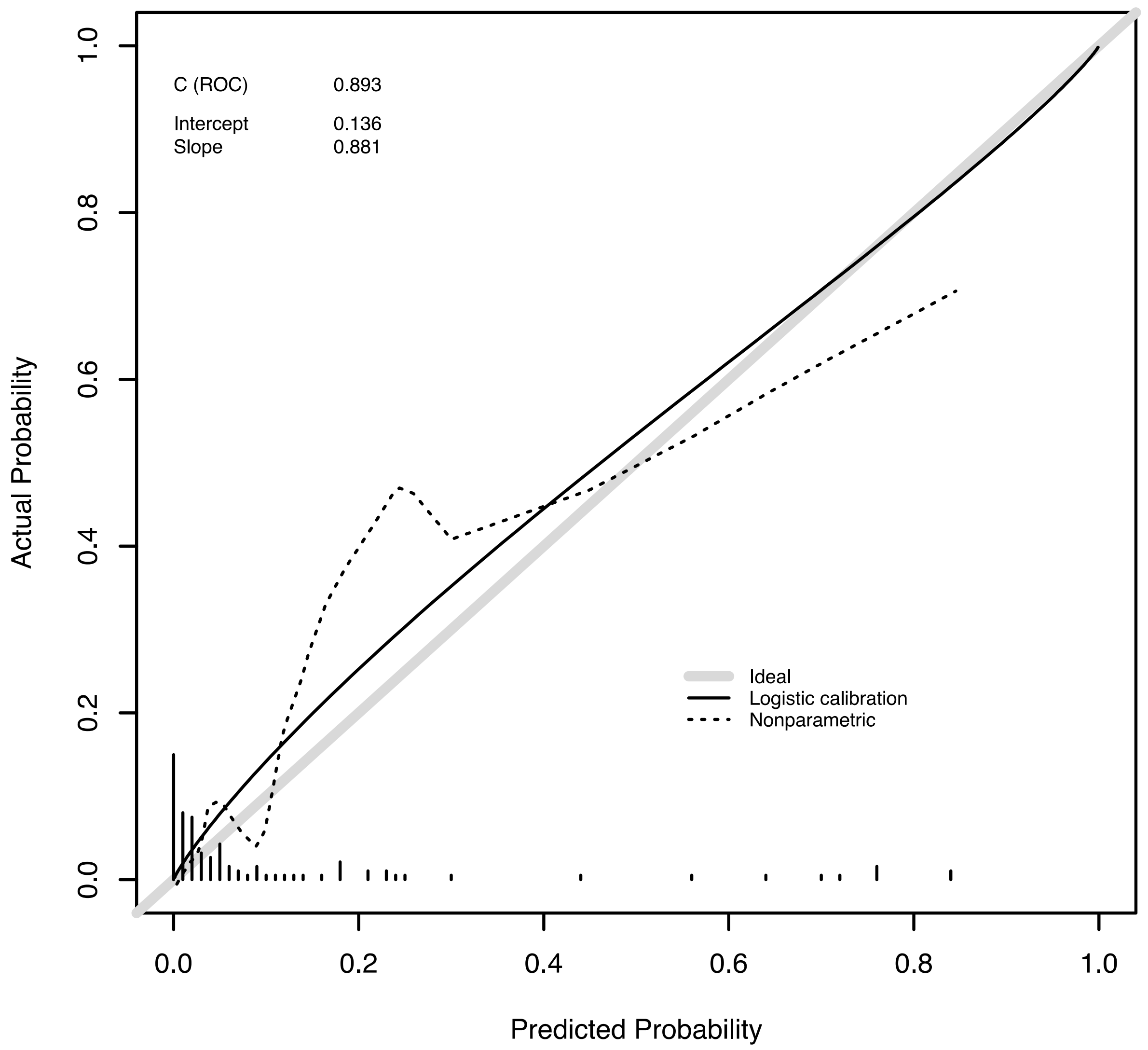

eFigure 5: Calibration Plot in Temporal Validation Cohort

Calibration plot showing actual *vs.* predicted probabilities of dependency in ADLs in temporal validation cohort. Ideal: model with perfect calibration, Logistic: calibration of original model, and nonparametric: calibration of original model in temporal validation cohort.

© 2022 Javeed S et al. *JAMA Network Open.*
