# Supplementary material for: Derivation and Validation of a Clinical Prediction Rule for Upper Limb Functional Outcomes After Traumatic Cervical Spinal Cord Injury
Source: JAMA Netw Open. 2022 Dec 21;5(12):e2247949. doi: 10.1001/jamanetworkopen.2022.47949 (PMC9857030; doi:10.1001/jamanetworkopen.2022.47949)
Supplement: Supplement 2. — Data Sharing Statement [file jamanetwopen-e2247949-s002.pdf]

Data Sharing Statement

Javeed. Derivation and Validation of a Clinical Prediction Rule for Upper Limb Functional Outcomes After Traumatic Cervical Spinal Cord Injury. *JAMA Netw Open*. Published December 21, 2022. doi:10.1001/jamanetworkopen.2022.47949

Data
Data available: Yes
Data types: Deidentified participant data, Data dictionary
How to access data: The de-identified participant data with a data dictionary defining each variable in the dataset is available from the SCIMS database website on reasonable request for legitimate research purposes.
https://www.nscisc.uab.edu/Research/NSCISC_DatabasePublicUse
When available: With publication

Supporting Documents
Document types: None

Additional Information
Who can access the data: Anyone requesting data
Types of analyses: Only for legitimate research purposes
Mechanisms of data availability: with a signed data use agreement
